# Interaction with a reactive partner improves learning in contrast to passive guidance

**DOI:** 10.1038/s41598-022-18617-7

**Published:** 2022-09-22

**Authors:** Ekaterina Ivanova, Jonathan Eden, Gerolamo Carboni, Jörg Krüger, Etienne Burdet

**Affiliations:** 1 Imperial College of Science, Technology and Medicine, London, SW7 2AZ UK; 2 Technische Universität Berlin, 10587 Berlin, Germany

**Keywords:** Biomedical engineering, Motor control

## Abstract

Many tasks such as physical rehabilitation, vehicle co-piloting or surgical training, rely on physical assistance from a partner. While this assistance may be provided by a robotic interface, how to implement the necessary haptic support to help improve performance without impeding learning is unclear. In this paper, we study the influence of haptic interaction on the performance and learning of a shared tracking task. We compare in a tracking task the interaction with a human partner, the trajectory guidance traditionally used in training robots, and a robot partner yielding human-like interaction. While trajectory guidance resulted in the best performance during training, it dramatically reduced error variability and hindered learning. In contrast, the reactive human and robot partners did not impede the adaptation and allowed the subjects to learn without modifying their movement patterns. Moreover, interaction with a human partner was the only condition that demonstrated an improvement in retention and transfer learning compared to a subject training alone. These results reveal distinctly different learning behaviour in training with a human compared to trajectory guidance, and similar learning between the robotic partner and human partner. Therefore, for movement assistance and learning, algorithms that react to the user’s motion and change their behaviour accordingly are better suited.

## Introduction

Which shared control strategy can ensure good performance and learning to drive a semi-autonomous car or assist a child on their first bicycle ride? To guide the user’s movement in robot-assisted applications, robotic interfaces traditionally use *trajectory guidance* (TG) with a spring-like force^1–3^. Such an interaction control strategy ensures accurate tracking of the reference trajectory, but can provide erroneous haptic information if the trajectory planned from the robot’s sensors is not appropriate for the task. Training assisted by guidance considerably changes the learners’ motion patterns by restricting movement freedom^4^ and, therefore, can induce passive behaviour that can hinder the learning as well as its generalisation after the assistance is removed^5–9^.

Could robot-assisted motor learning be improved by incorporating the strategies used by humans during shared control? Humans routinely interact with each other e.g. to carry large objects together or during dancing. Although during such joint tasks partners communicate only by the exchange of forces, they can swiftly coordinate motions and adjust their movements to the partner. Recent studies investigated such haptic interaction in pairs of subjects connected by an elastic band and carrying out a tracking task^10,11^, which revealed that *human partners* (HP) conspicuously exchange sensory information to improve their own performance^12–14^. Specifically, the benefits of haptic interaction in joint performance were revealed^10^ and subsequently shown to stem from the exchange of sensory information between the partners enabled by the haptic channel^12^. The *robotic partner* (RP) introduced in^12^ to embody this haptic communication hypothesis was shown to provide similar performance and perception as human partners^4^.

The benefits of interactive control with a HP and a RP suggest that it may be used to boost performance in collaborative tasks such as shared driving, rehabilitation training and joint object manipulation. However, it is still unclear whether learning with a human or robot partner would offer any advantage over training alone. The previous publications studying human–human interaction report conflicting results on the effect of this type of motion assistance on learning. Some studies have reported benefits of human–human interaction on learning^10,15^, while in other studies no significant differences in performance were observed^11,16^, or it was found that learning strongly depends on the partners’ skills during the interaction^17^. More importantly, these previous studies have only looked at the subject’s change in performance directly after the training session or at the differences within training, while sensorimotor performance can change with time^18^, thus it is crucial to assess motor performance after several days^19^.

Most studies of human assisted learning concern some type of trajectory guidance^9,20–23^ where the results show that a learner usually relies on such assistance during the training, which results in reduced learning transfer when the connection with the assistance is removed. Only a few compliant or reactive robotic algorithms such as fading haptic guidance^9^ and model predictive control^24^ have been proposed and investigated in terms of learning. Moreover, the learning provided by robotic interaction control has been extensively investigated for reaching arm movements^25–27^, which are carried out largely according to a predefined plan^28,29^. For tracking tasks in which incoming sensory information must be continuously used, little research has considered the effects of robotic interaction.

This paper reports the first study of long-term learning in human–human interaction. We designed an experiment to analyse and systematically compare the tracking performance, learning and retention that resulted from training with a human or the human-like robot partner controller of^12^ relative to training with trajectory guidance. Moreover, we compare the resulting learning in all interactive conditions to training without interaction (the “solo” condition).

**Figure 1:**
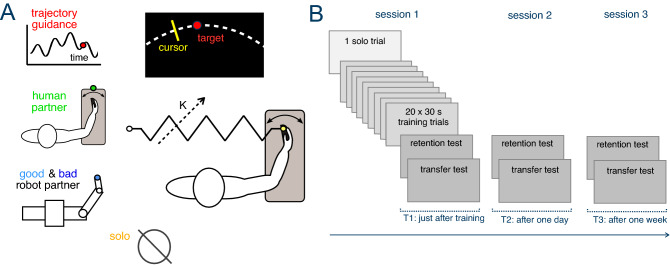
Experiment description. (**A**) Subjects use the Hi5 robotic interface to track a randomly moving target with their wrist flexion/extension. This occurs either without interaction (S) or while connected to: trajectory guidance (TG), their human partner (HP), or a reactive robot partner with less deviation (RPg) or more deviation (RPb) than the subjects’ initial error. (**B**) Experimental protocol with one solo performance test trial followed by 20 learning trials connected to one of the partners at the selected stiffness level. Performance after training was tested immediately after learning (T1), one day (T2) and one week (T3) later.

**Figure 2:**
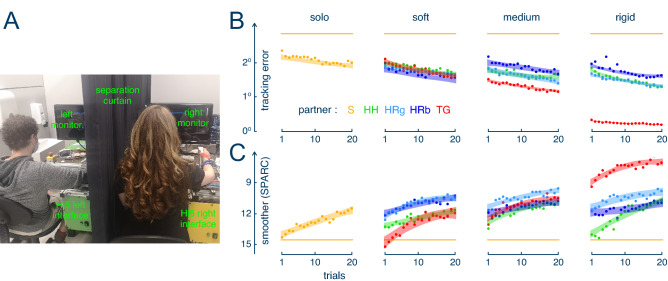
(**A**) Experimental setup for the tracking task: participants were separated by a curtain and tracked a visual target presented on the monitor using handles of the dual robotic interface Hi5. (**B**,**C**) Learning analysis using the tracking error (**B**) and the SPARC smoothness metric (**C**). The different connection conditions were: “solo” (no connection), “soft” 0.29 Nm/rad stiffness, “medium” 1.72 Nm/rad, “rigid” 17.02 Nm/rad. The dots represent the average of the corresponding metric for a trial and the area around it represents the ± 95% confidence interval estimated based on the least square fit of a second order polynomial. The horizontal yellow bars correspond to the metric’s value in the initial trial. Figures 2(**B**,**C**), 3, 4, 5 were created using RStudio (Version: 1.2.1335; https://www.rstudio.com/).

Pairs of subjects, separated by a curtain preventing visual communication, moved their individual handle of the Hi5 dual robotic interface^30^ to control a cursor on their own monitor (Figs. 1A, 2A). Each subject was required to track a target moving along a multi-sine function “as accurately as possible” using wrist flexion/extension of their dominant hand (see “Methods”). During the tracking task, subjects were connected to a partner agent by an elastic spring, where the partner angle would depend on their type. Four interaction conditions were used in our experiments: *human partner* (HP), *“bad”* or *“good” robot partner* (RPb, RPg) with 40% lower and 40% higher accuracy than initial subjects’ performance respectively, *trajectory guidance* (TG), as well as and three stiffness levels: *soft* (0.29 Nm/rad), *medium* (1.72 Nm/rad), *rigid* (17.02 Nm/rad), yielding twelve different experimental conditions, complemented by a *solo* condition (S) in which subjects performed the task without connection. 180 participants trained in one of these 13 conditions following the protocol of Fig. 1B. One familiarisation trial without interaction preceded 20 training trials with the specific human or robot partner and connection stiffness. Learning was assessed immediately after this training, one day later and one week later. Retention was evaluated in one trial with the same task as in the training, followed by one 30 s long trial with a different multisine target trajectory to test the transfer of learning (see “Methods”).

As interacting with a human partner will provide additional sensory information during interaction^12^, our first hypothesis (H1) is that this will lead to better performance and learning than when training without interaction. We further assumed that the robot partner, based on such sharing of sensory information^12^, will influence the learning in the same way as a human partner (H2). Finally, we anticipated from previous studies^8,9^ that trajectory guidance can impede learning when the interaction link is removed compared to training alone (H3).

## Results

### Impact of interactive control

We analysed the subjects’ performance during the training phase while being connected to one of the interaction agents, as well as how the performance changed over the course of the training. In every partner and stiffness condition, the tracking error and error variability decreased from the first connected trial relative to the familiarisation trial that was performed solo (all p < 0.0001) (Fig. 2B). Smoothness improved in the first training trial when interacting with both the RPg and the RPb at every stiffness level (all p < 0.02) (Fig. 2C). For the TG this strongly depended on the connection stiffness, where the TG with the medium and rigid stiffness immediately increased smoothness (t(308) = − 2.533, p = 0.028 for the medium, t(308) = − 13.272, p < 0.0001 for the rigid connection), but no such effect was observed for the soft stiffness condition (t(308) = 0.106, p = 0.980). However, motion smoothness was not immediately improved while connected with a HP (p > 0.23 for each stiffness values) or while conducting the task alone (t(13) = 8.568, p = 0.325).

The tracking error decreased in most conditions during the 20 training trials based on the difference between the first and the last five connected trials (all p < 0.05) (Fig. 2A). One exception was the HP condition with medium stiffness, which did not improve the subjects’ tracking accuracy during the training (t(308) = 2.036, p = 0.073). Another exception was the connection to TG with the rigid stiffness (t(308) = 2.036, p = 0.299), however, this condition resulted in a more accurate performance compared to the other groups from the first connected trial (all p < 0.0001). Even though the accuracy increased during the 20 connected trials, the error variability reduced in only some conditions: while connected to RPs at the medium stiffness (RPg: t(308) = 2.721, p = 0.017; RPb: t(308) = 2.323, p = 0.048), while working with TG at the soft and medium stiffnesses (soft: t(308) = 3.632, p = 0.001; medium: t(308) = 2.498, p = 0.031) and while completing the task with a HP at rigid stiffness (t(308) = 3.632, p < 0.0001). Learning was also indicated through a large increase of smoothness during the 20 trials for all partner and stiffness conditions including the solo condition (all p < 0.02), except for the RPb group with medium (t(308) = − 1.952, p = 0.1197) and rigid stiffness (t(308) = − 1.568, p = 0.221) (Fig. 2B).

### Adaptation to shared control

**Figure 3:**
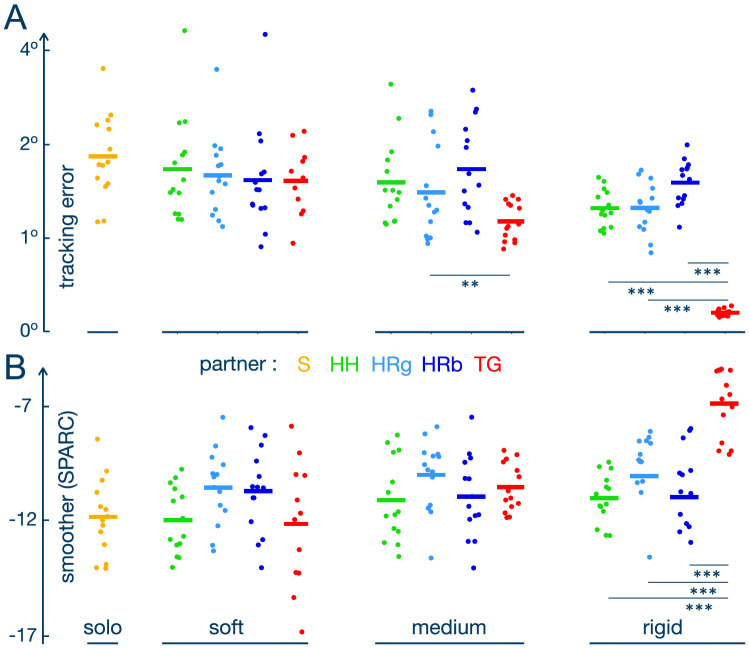
Tracking performance and smoothness with the partner’s assistance at the end of the training period. Each dot represents the average tracking error (**A**) and SPARC smoothness metrics (**B**) in the last five trials for one participant. The bars show the average value for each condition.

At the end of the training, all groups exhibited similar error levels for the soft connection (p > 0.59 for all pairwise comparisons for the averaged value over the last five trials) (Fig. 3A). However, clear differences appeared for the other stiffness levels. For the medium connection, subjects from the TG group followed the target more accurately than the HP (t(243) = − 2.260, p = 0.044), RPb (t(167) = − 3.032, p = 0.006) and S (t(167) = − 5.306, p < 0.0001) groups. Furthermore, the RPg group showed less error than participants tracking the target solo (t(167) = − 3.247, p < 0.014). With the rigid connection, the TG resulted in a higher accuracy than all other conditions (all p < 0.0001). Moreover, while rigidly connected, the HP and RPg conditions resulted in higher accuracy than conducting the task solo (S > HP: t(167) = − 4.371, p = 0.0003, S > RPg: t(167) = − 4.347, p = 0.0003).

As expected, the TG decreased the error dramatically with a stiffer connection: the error was lower with the medium stiffness compared to the soft connection (t(308) = − 2.254, p = 0.044) and with the rigid stiffness compared to less stiff connections (both p < 0.0001). In contrast, the error was relatively insensitive to the connection stiffness in the RPg and RPb groups (p > 0.097 for all pairwise comparisons between different stiffness levels for each group). Finally, the accuracy during the final trials with the HP was similar between the rigid and medium as well as between the soft and medium stiffness levels (both p > 0.2). However, the accuracy was lower for the soft compared to the rigid connection (t(308) = 2.273, p = 0.044). The average error for the HP tends to be as low as with the RPg over all stiffness conditions (t(154) = 0.321, p = 0.999).

A similar pattern was observed for the error variability at the end of the training: TG showed less variability than the HP (t(266) = − 2.721, p = 0.017), RPb (t(266) = − 2.843, p = 0.013) and S (t(167) = − 5.165, p < 0.0001) conditions with medium stiffness and less variability than all other conditions with rigid stiffness (all p < 0.0001). Furthermore, the RPg resulted in less error variance than S with medium (t(167) = − 3.652, p = 0.004) and rigid (t(167) = − 4.477, p = 0.0002) connections. Interaction with a HP was only different from S for the rigid stiffness (t(167) = − 4.228, p = 0.0004). No differences between the partner conditions were found for the soft connection (all p > 0.24), and the HP, RPg resulted in a similar variability for all connection levels (t(154) = 0.978, p = 0.909).

Roughly symmetric changes were observed for the last five trials on the smoothness (Fig. 3B). As expected, movements with the TG had the highest smoothness after training in the rigid condition compared to the other partner groups and solo condition (p < 0.0008 for comparison with each partner condition for rigid stiffness level inclusive control group). Moreover, the smoothness for the TG was higher for the rigid stiffness than for the medium (t(255) = 4.372, p = 0.0001) and soft connections (t(255) = 6.050, p < 0.0001). Although in the first connected trial for the soft connection, movements with the TG were not particularly smooth relative to the RPs, at the end of the training no differences between these partner conditions were identified (all p > 0.13). While at the beginning of the training with the rigid stiffness, interaction with another human resulted in the lowest smoothness among other condition, the differences between the HP and RPs disappeared after 20 trials (all p > 0.3). No other differences between the groups were revealed for the last five connected trials (all p > 0.05).

### Acquired behaviour in visuomotor tracking

**Figure 4:**
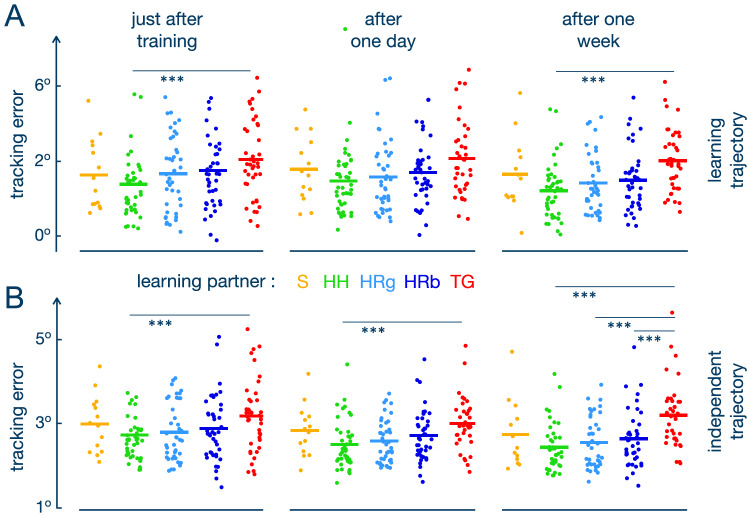
Retention (**A**) and transfer (**B**) of the tracking performance without interaction after learning. Each dot represents the value of mean tracking error in the same trajectory used during training (**A**) and in an independent multisine trajectory (**B**). The bars show the average value for each condition.

To evaluate the effect of learning on individual performance, we analysed the influence of the stiffness and partner conditions on the accuracy, error variability and smoothness in the retention and transfer trials. Figure 4A shows the tracking error using the training trajectory in the three retention trials — immediately after training, after one day and after one week. The analysis showed a significant effect of the partner condition on the retention accuracy (F(3,153.45) = 4.027, p = 0.009). We observed that training with the TG resulted in a larger error than training with a HP over all stiffness levels and trials (t(154) = 3.408, p = 0.005). A small difference was seen between TG and RPg groups, however, this comparison was not significant (t(154) = 2.317, p = 0.065). The tracking error for the TG with the rigid stiffness was also larger relative to the S condition (t(167) = 3.649, p = 0.003) over all retention trials.

Error variability was also significantly impacted by the partner condition (F(3, 153.17) = 5.377, p = 0.0015). Subjects that trained with TG had significantly more variability in their retention trials than those with a HP (t(154) = 3.950, p = 0.0007) and the RPg (t(154) = 2.508, p = 0.0396) groups over all stiffness levels and retention trials. Moreover, the HP group showed less error variability in retention than S over all stiffness conditions (t(175) = − 2.474, p = 0.0377).

The retention smoothness did not change for different partner conditions (F(3, 154.34) = 1.487, p = 0.220), but was significantly influenced by the connection stiffness (F(2, 154.35) = 3.176, p = 0.045). Participants that trained solo had smoother movements in retention than those that trained with the rigid connection (t(176) = − 2.287, p = 0.047). The rigid stiffness also tended to have lower smoothness than the soft (t(154) = − 2.059, p = 0.062) and medium connections (t(154) = − 2.282, p = 0.062), however these results were not significant.

Clearer differences between the groups were observed along the independent trajectory used to infer the transfer of learning (Fig. 4B): the partner condition (F(3, 153.53) = 5.551, p = 0.001) as well as its interaction with the test time (F(6, 299.10) = 2.745, p = 0.013) had a significant effect on the tracking error in the transfer trial. In particular, the TG resulted in more error than all of the other groups after one week (HP<TG: t(195) = − 4.855, p < 0.0001; RPb<TG: t(198) = − 3.333, p = 0.006; PRg<TG: t(198) = − 4.016, p = 0.0008). Moreover, even immediately or one day after training the subjects that trained with the TG conducted their movements less accurately than those that trained with a HP (immediately: t(194) = − 2.883, p = 0.016; after one day: t(198) = − 3.063, p = 0.011) and than those in the RPg condition (immediately: t(194) = − 2.436, p = 0.041; after one day: t(199) = − 2.474, p = 0.041). The TG condition also had a larger error than the S condition for the rigid stiffness over all transfer trials t(167) = 3.291, p = 0.011). In contrast, the HP condition had a lower error than the S over all stiffness levels and retention trials (t(175) = − 2.475, p = 0.037).

The error variability in transfer was also influenced by the partner condition (F(3, 152.24) = 3.800, p = 0.0116) and its interaction with the test time (F(6, 298.08) = 2.8681, p = 0.0099). The differences in variability between partner conditions were observed only in the transfer trial after one week: TG showed more variability than the HP (t(217) = 4.199, p = 0.0007), RPb (t(220) = 3.135, p = 0.0117) and RPg conditions (t(220) = 3.673, p = 0.0027).

The transfer smoothness, similar to the retention smoothness, was influenced by the stiffness (F(2, 154.32) = 4.512, p = 0.012) and its interaction with trial (F(4, 300.33) = 3.216, p = 0.013). Training with the rigid connection resulted in lower smoothness than with the soft stiffness in the first retention trial (t(227) = 3.299, p = 0.008) and then with the medium stiffness after one day after training (t(232) = 3.152, p = 0.008).

## Discussion

Our experiment investigated how subjects perform a tracking task with wrist flexion/extension on a relatively complex trajectory. This task demands continuous planing based on incoming sensory information, in contrast to the widely studied reaching arm movements that can be carried out largely according to an initial plan^28^. By comparing representative interactive controllers with regards to performance and learning, we analysed their ability to assist movement for shared control, and the tracking performance they induce. This study is the first to analyze the long-term learning effect of training with a human partner (HP) and with the robotic partner (RP) of^12^ up to one week of retention, compared to solo performance (S) and training with trajectory guidance (TG).

During the shared control stage, when the subjects were connected to one of the partner conditions, the TG, HP and RPg all reduced the tracking error significantly relatively to the S condition when the connection between the partners was rigid. However, the manner with which the TG does so is distinctively different to the HP and RP conditions. The TG uses an elastic force to the known desired trajectory and thus results in more accurate tracking with increased connection stiffness. This results in a nearly perfect accuracy from the first connected trial, dramatically decreases error variability, and leads to higher smoothness. As a consequence, assistance from TG induces a considerable change of motion patterns compared to what a learner would exhibit during solo training. In contrast, the HP and RP do not assume a-priori knowledge of the planed trajectory as they predict it^12^. The HP’s or RP’s movement is different from the target trajectory and does not decrease with a more rigid connection. Moreover, the motions characteristics with HP and RP are similar to the solo condition, which indicates that these reactive agents help improve performance *without changing the behaviour*.

**Figure 5:**
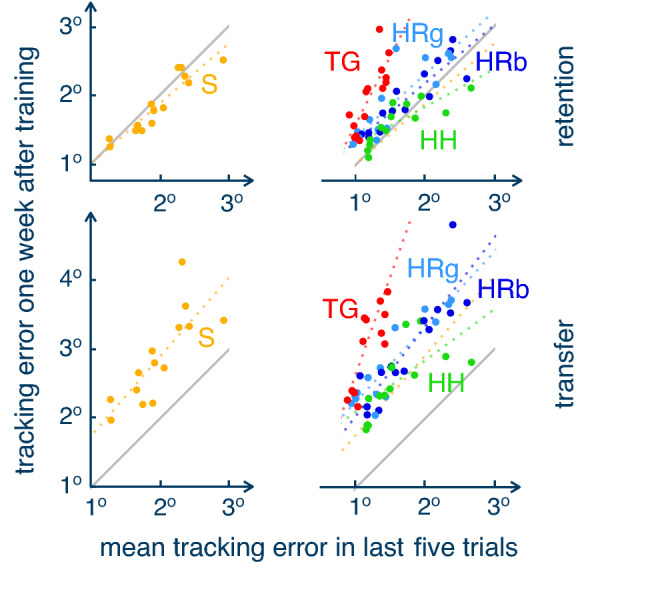
Training tracking accuracy retension/transfer relationship. Accuracy is taken from the last five trials of training while the average performance in transfer and retention trials at medium stiffness is used. Dashed lines represent the least-square fit for each group. The grey line separates the graph so that in the upper triangle it contains participants with better performance just after training than one week later, while in the bottom triangle it has subjects who improved performance one week after training.

The effect of training with different interaction control modalities also separates the TG from the HP and RP as can be observed at the end of the training period, as is visualised in Fig. 5. The TG minimises the tracking error, error variability and smoothness while the connection is maintained. However, after it leads to deteriorating tracking performance as is particularly observed one week after training (Fig. 4B). This can also be observed from Fig. 5 where the TG had the stiffest slope in retention (b = 1.9639, t(12) = 4.894, p = 0.0004) and in transfer (b = 2.8225, t(12) = 3.542, p = 0.0041). Moreover, regression analysis revealed that the TG slope was different from all other groups in retention (all p < 0.027) and from HP and RPg in transfer (both p < 0.45). This observation confirms the H3 hypothesis and previous findings, where despite improved training performance during connection, haptic guidance impedes learning and its generalisation in path-following^31^, continuous rhythmic^9^ and timing-crucial^6^ tasks as well as to control an unstable inverse pendulum^24^.

Interestingly, the performance after training solo remains stable after one week (Fig. 4A), arguably corresponding to learned task performance. Similarly, performance after one week did not deteriorate further after training with the RPs, where statistical comparison between the S and RP groups also did not exhibit a significant difference for the regression slopes. The HP, in contrast and in accordance with the H1 hypothesis, did show a tendency to improve after training relative to the S condition (Fig. 4B), which resulted in a more flat slope in Fig. 5 than other groups in retention (b = 0.5457, t(12) = 4.615, p = 0.0006) and transfer (b = 0.7387, t(12) = 3.210, p = 0.0076). These results might have been affected by the use of healthy subjects in a one-dimensional task and therefore need to be investigated further.

In this study we compared robot partners with high and low noise, which are different parameterisations of the RP, where the level of accuracy that the partner provides was varied. In this way we could test how the skill level of this human-like robot partner influenced learning. The RPg condition tended to lead to better performance during the training compared to the other conditions, however, after the training this effect disappeared and both RPs have a similar retention and transfer of the learned skills. The similarity of performance in retention and transfer regardless of the quality of the RP, suggests that for continuous tasks the accuracy of the robot partner might be not as important factor as the manner of how the partner interacts characterised by the intrinsic reactivity of the controller or its compliance. Since there is no difference between the robotic skill partner level and the learning with RPs is superior to TG, it is clear that assistance corresponding to the user’s ongoing movement is preferable over assistance through trajectory guidance, which agrees with recent results comparing compliant predictive control with TG^24^.

While tracking error and error variability are mostly influenced by the interaction modality during the training phase, motion smoothness in retention and transfer depended on the connection stiffness of the training partner. Regardless of the partner condition, training while connected through a rigid link impacted the smoothness negatively compared to training solo or with the soft and medium stiffness levels. This may be linked to the rigid stiffness reducing the subject’s ability to freely move within the training, thereby not providing them with the opportunity to find more naturally smooth motions.

In summary, shared control with a human partner or the robotic partner of^12^ leads to a reduction of the tracking error when moving together and increases the movement smoothness without impacting the ability to perform the task when alone or reducing some error variability in the motion. With the exception of working with a HP, no partner showed clear improvements in the retention or transfer trials compared to the S condition, where in particular TG resulted in reduced performance, which is likely due to slacking behaviour as was suggested in previous studies^5,7^. This may be explained by the fundamentally different mechanisms of interaction: while TG physically guides the learner and restricts their own motion flexibility especially when the connection between partners is rigid as was shown in^4^, the HP and RP benefit performance and learning by providing additional sensory information without interfering with the intended motion. Due to TG modifying behaviour, e.g. by invoking passive performance, and its inability to deal with intrinsic human variability, TG is only suited to applications where the human cannot perform the task actively, as in robot-aided stroke rehabilitation for severely affected individuals. Instead, in applications such as active neurorehabilitation, shared driving^32^ or co-pilot systems^33^, an interaction with another human or RP is better suited. Furthermore, when learning is required, despite being widely used, the negative impact of TG on learning means again a HP or RP is more appropriate.

Finally, while the RPs generally exhibited similar behaviours to the HP condition during and after training, they present differences that are worth analysing. While a connection with RPs immediately improved the subjects’ smoothness in the first training trial, interaction with a HP did not show this effect. Participants interacting with a HP also did not, in the medium stiffness condition, improve their accuracy during the training phase, while clear improvements were visible in all RPs conditions. Therefore, during the training the RPs showed a better performance than HP. It is however important to highlight that the HP was the only condition that showed a better accuracy than S in retention and transfer. This suggests that, contrary to the H2 hypothesis, there are unique characteristics of this human interaction that specifically possess the ability to improve motor learning. Identifying these characteristics and how to replicate them in human-like robot controllers as well as further generalisation of these results to robotic interfaces with higher degree-of-freedom and other tasks is therefore critical.

## Conclusion

We evaluated different mechanisms for robot-assisted training. The results show that interaction with another human or with a human-like robot partner improves performance during interaction and learning up to one week. In contrast, trajectory guidance, which modifies the learner’s behaviour considerably during interactive training, does not provide efficient learning. This suggests that a human-like robot partner is a suitable controller for automated training, since it enables the exchange of sensory information without interfering/restricting the motion during interaction.

## Methods

### Participants

The experiment was granted ethical approval by the Research Ethics Committee of Imperial College London (reference 15IC2470). The study was performed in accordance with all relevant guidelines and regulations. 180 healthy volunteers (66 females and 134 males, aged 17–41 years with an average age of 24.2 and standard deviation of 3.8) took part in this study. Twelve participants were left-handed, 167 right-handed and one ambidextrous. 129 participants reported some experience with haptic devices such as gaming controllers or joysticks and 138 regularly play or used to play computer games (from 0.1 to 40 h/week with a mean of 7.6 h and standard deviation of 7.6 h). 19 participants reported previous practice with a robotic interface.

### Experiment setup and procedure

Before beginning all participants gave their informed consent to carry out the experiment, then filled in the Edinburgh handedness form^34^ and a demographic questionnaire. They were instructed that within the experiment they might interact with a robot, another human, or complete the training without interaction. Subsequently, they were randomly assigned to one of the thirteen experimental groups. Altogether each group had 14 subjects, with the exception of the TG group with the soft stiffness connection which had only twelve participants.

Subjects were seated in front of a monitor with their dominant hand connected to one of the handles of the Hi5 robotic interface. They used their hand to track the target trajectory shown on the screen, which was given (in degrees) by

1 $$\begin{aligned} q^*(t) \equiv \, 18.5 \, \sin \! \left(2.031\,\,t \right) \, \sin \!\left(1.093\,\,t \right) \,, \quad 0 \le t \le 30\,s. \end{aligned}$$

The interface yielded an elastic connection of the wrist flexion/extension *q*(*t*)

2 $$\begin{aligned} \tau (t) \equiv \, \kappa \left[ q_r(t)-q(t) \right] \,\, \text{Nm}, \quad 0 \le t \le 30 s, \end{aligned}$$

with the reference angle $q_r(t)$, which was differently set for each experimental condition, during 30 s long trials. The connection stiffness $\kappa$ was set as one of {0.29, 1.72, 17.02} Nm/rad, since this parameter has been shown to impact the interaction behaviour as was detailed in^4^. The Hi5 was operated in torque control, and enabled the interaction at 1000 Hz. Wrist angle data was simultaneously recorded at 100 Hz.

All subject pairs participated in three sessions as shown in Fig. 1B. On the first day, they completed one test trial without any interaction torque, followed by one of the interaction conditions for 20 30 s long trials with 10 s breaks in between. Learning was assessed immediately after training, after one day, and after one week. Each of these assessment consisted of one *retention trial* without interaction, followed by one *transfer trial* on a different trajectory given by

3 $$\begin{aligned} q^*(t) \equiv \, 22.2 \, \sin \! \left(\,2.031\,(t+1.2)  \right) \, \sin \!\left(\,1.093\,(t+1.2)\right) \,, \quad 0 \le t\,\le \, 30\,s. \end{aligned}$$

### Experimental conditions

The 13 experimental groups corresponded to one control group that performed the complete experiment without working with a partner plus groups for each combination of partner agent {HP, TG, RPg, RPb} and stiffness level {0.29, 1.72, 17.02} Nm/rad. Each subject completed the training in only one of the 13 condition (between-subjects study design).

For each different partner type the reference angle $q_r$ was set differently. In the *trajectory guidance (TG)* condition, the subject performed the experiment connected to the reference trajectory such that $q_r = q^*$. The interaction force (2) therefore acted as a proportional position controller.

In the *human partner (HP)* condition, two subjects simultaneously performed the tracking with trajectory (1) by holding their respective robotic interface with a virtual spring connection between them, such that $q_r$ was given by their partner’s position. The subjects were not given explicit knowledge that they were working together but they were given indirect knowledge of their partners position through the interaction force (2).

Finally in the *good and bad robot partner (RPg and RPb)* conditions, the subjects were connected to a robot agent that tracked the measured target using the human-like partner algorithm of^12^. Here, the agent evolves $q_r$ using a control input which is given by the linear feedback control law

4 $$\begin{aligned} u = \,-\, L_p \! \left( q - \widehat{q^*}\right) \, + \, L_v \! \left( {\dot{q}} - \widehat{{\dot{q}}^*}\right) , \end{aligned}$$

where $L_p$ and $L_v$ are the proportional and derivative gains and $\widehat{q^*}$ denotes the RP’s target estimation. To obtain this target estimate, the RP combines its own measurement of the target with the partner’s target estimated from the interaction force between them. This is achieved using a Kalman filter that uses the known dynamic model and a measurement consisting of both the partners own measurement and its estimation of the partner. The good and bad partners are achieved by altering the amount of noise in the RP’s partner estimation. The RPb was set so that the resulting *tracking error*

5 $$\begin{aligned} \frac{1}{T} \! \int _0^T \!\!\! \left| \, q(t) - q^*\!(t) \, \right| \, dt\,, \quad T = 30 s, \end{aligned}$$

where $q^*$ is the target position. Two different RPs, “good” and “bad”, were considered which used different noise in their partner estimation: RPb had a 40% higher error compared to the subject’s initial performance and the RPg was set to 40% lower error.

### Statistical analysis

Performance and learning were analysed using the *tracking error* defined by Eq. (5), the *error variability* defined as a characteristic of motion variability, calculated as a standard deviation of errors during each trial duration, as well as the *SPARC smoothness* metrics^35^ that evaluates the complexity of the movement velocity ${\dot{q}}$. For all metrics the first 0.8 s of each trial was deleted to exclude the reaction time at the movement start and to analyse only the tracking performance. Metrics were analysed during the connection with a partner to estimate shared performance and after the link between partners was removed to evaluate the resulting acquired skills.

To examine the subjects’ initial skills level, we analysed the smoothness and accuracy using a 2-way ANOVA with two in-between predictors — partner and stiffness. We conducted tailored post-hoc comparisons using t-test contrasts to investigate the differences between the single factor levels. Moreover, a Dunnett test was conducted to compare each condition to a control group. The Benjamini–Hochberg adjustment was used to control the false discovery rate resulting from multiple comparisons. Since all 13 groups were not different in the performance (all p > 0.06), in following analysis this skills baseline was not considered.

To investigate performance during the training we conducted a three-way mixed ANOVA with two in-between factors — partner and stiffness condition, and with one repeated measures predictor — training trial. We used tailored t-test contrasts with Benjamini–Hochberg adjustment to investigate the difference between the groups in the last five trials and within the same groups between different training trials. The differences between different trials in the solo group were analysed with paired t-tests. The comparisons between the S and other groups were realised using Dunnett tests.

To investigate how training with shared control influenced the learning, we analysed the smoothness and accuracy in the retention and transfer trials immediately, one day and one week after training. Due to the presence of missing observations in the data for some of the post-tests and non-normal data distribution in some of the conditions, we conducted an ANOVA using a linear mixed-effects model. For all dependent variables, we fitted a model with fixed effects on the stiffness, partner condition, test time (immediately after training, one day or one week after) and their interactions, and the random intercepts for the subject number. When one of the factors or their interaction was significant, post-hoc comparisons using t-test contrasts with a Benjamini–Hochberg adjustment were employed. A Dunnett test was conducted to compare each condition to the control group.

## Supplementary Information

Supplementary Information.

## Data Availability

All data generated or analysed during this study are included in this published article and its supplementary information files.
